# Demonstration of a Gandolfi-type attachment for fast high-resolution synchrotron XRD of non-ideal specimens

**DOI:** 10.1038/s41598-026-43550-4

**Published:** 2026-03-12

**Authors:** Shintaro Kobayashi, Shogo Kawaguchi, Yuki Mori, Kei Watanabe, Michitaka Takemoto

**Affiliations:** 1 Japan Synchrotron Radiation Research Institute, 1-1-1 Kouto, Sayo, Hyogo 679-5198 Japan; 2 Department of Life Science, Graduate School of Science, University of Hyogo, 2167 Shosha, Himeji, Hyogo 671-2280 Japan

**Keywords:** Materials science, Physics

## Abstract

Synchrotron powder X-ray diffraction (PXRD) offers significant advantages in the structural analysis of functional materials and enables the acquisition of high-quality data; however, accurate data collection remains challenging for samples consisting of coarse crystallites or molten samples. Specifically, obtaining reliable PXRD data from samples in their as-solidified or non-pulverized state remains challenging during melting–solidification and crystal grain growth processes, as well as for materials produced by these processes. To mitigate these limitations, a two-axis rotation Gandolfi-type attachment—comprising a 45°-tilted *φ*-axis and its rotational *ω*-axis—was developed and implemented on a high-resolution powder diffractometer at SPring-8, which is equipped with fast area detectors. This configuration improved the particle statistics by increasing the number of crystallites satisfying the Bragg condition through two-axis rotation, while also stabilizing the sample position, even for molten samples, owing to the tilted geometry. Specifically, the integration of a high-speed spinner and multiple two-dimensional photon-counting detectors allowed sub-second continuous imaging and frame-by-frame peak separation, facilitating the indexing of PXRD data for complex structures. The analytical capability was evaluated in three case studies, namely the pair distribution function analysis of molten Zn, in situ observations of LiCoO_2_ electrode material synthesis in a molten flux, and high-resolution PXRD of mineral crystals within a short timeframe. The results confirmed that the Gandolfi-type attachment improved data quality and reproducibility, thereby enabling reliable measurements even for practical samples with limited availability of fine powders.

## Introduction

The advancement of functional materials for sustainable and energy-efficient technologies requires reliable and precise sample characterization methods. Among various analytical methods, powder X-ray diffraction (powder XRD, PXRD) techniques are widely used to characterize materials by the determination of constituent phases, crystal structures, and volume fractions, as well as by tracking in situ structural changes under a range of external conditions. Synchrotron XRD is particularly advantageous because of its ability to rapidly acquire high-quality XRD data by utilizing high-brilliance, high-energy X-rays^1,2^. In recent years, the introduction of hybrid photon-counting detectors has promoted the development of high-throughput measurements^3,4^. Although measurement systems have become increasingly advanced, precise quantitative analyses still require fine powder samples to obtain good-quality PXRD data with sufficient particle statistics. In other words, accurate quantitative analysis is often difficult for samples composed of single crystals or coarse crystallites, because they typically produce few diffraction peaks and fail to yield ideal intensity ratios in the PXRD patterns.

One practical approach for addressing these challenges is the use of a Gandolfi camera layout^5–13^, comprising a 45°-tilted *φ*-axis and its rotational *ω*-axis. This two-axis rotation allows a wide range of crystal orientations to satisfy the diffraction conditions, thereby enabling the acquisition of high-quality PXRD data, even from limited sample quantities. Gandolfi cameras have been widely used in mineralogy and implemented in various laboratory instruments and synchrotron radiation facilities^6–11^. Imaging plates have often been employed as two-dimensional (2D) detectors in Gandolfi cameras to collect numerous diffraction spots; however, this approach has inherent limitations, such as long acquisition times and short sample-to-detector lengths, which restrict the throughput and achievable angular resolution. To address these limitations, Tanaka et al. developed a large Gandolfi camera with a camera length of 955 mm at a synchrotron facility^12^. However, further improvements are still necessary, particularly with regard to expanding the angular coverage attainable within a short exposure time.

To overcome these limitations, the implementation of a Gandolfi camera system equipped with fast-readout 2D detectors is expected to enable the acquisition of data with high statistical accuracy within a short measurement time. A suitable platform for such a system is the high-resolution powder diffractometer that was recently introduced at the BL13XU beamline of the SPring-8 facility, which is permanently equipped with multiple 2D photon-counting detectors and a large-area flat-panel detector (FPD)^14^. Notably, the adoption of a Gandolfi camera layout combining high-brilliance synchrotron X-rays and fast area detectors enables the acquisition of high-precision data within a few minutes. Establishing such a rapid acquisition system would significantly broaden the applicability of synchrotron PXRD, enabling measurements during in situ heating and cooling processes where melting, solidification, or grain growth occurs. In addition, the high-throughput characterization of bulk materials that cannot be readily pulverized into fine powders suitable for conventional powder diffraction measurements would become feasible. This approach also allows the straightforward characterization of systems in which pulverization degrades intrinsic structural information, thereby supporting efficient sample evaluation and accelerating materials development. Realizing such a rapid acquisition system requires the design of a Gandolfi camera layout capable of rotating target samples at high speeds, which would maximize the performances of high-brilliance synchrotron X-rays and fast area detectors.

Herein we report a Gandolfi-type two-axis rotation attachment incorporating fast area detectors for the accurate acquisition of PXRD data within a short timeframe. Specifically, the availability of multiple photon-counting detectors is expected to enable the rapid acquisition of low-background and high signal-to-noise PXRD data. To accommodate the short exposure times, a high-speed spinner is combined with the fast area detectors to enable sub-second continuous imaging with frame-by-frame peak separation. This should facilitate the efficient indexing of complex crystal structures. Furthermore, PXRD measurements are performed using a 45°-tilted spinner that stabilizes the position of the liquid phase in the capillary under the influence of gravity. This configuration is expected to improve the reproducibility of in situ heating experiments for synthesis in molten salts, as well as enhance the reliability of pair distribution function (PDF) analysis during melting. Finally, the applicability of the proposed PXRD system with two-axis rotation is investigated for a wide range of sample types.

## Results and discussion

### Two-axis rotation attachment for precise data acquisition

**Fig. 1:**
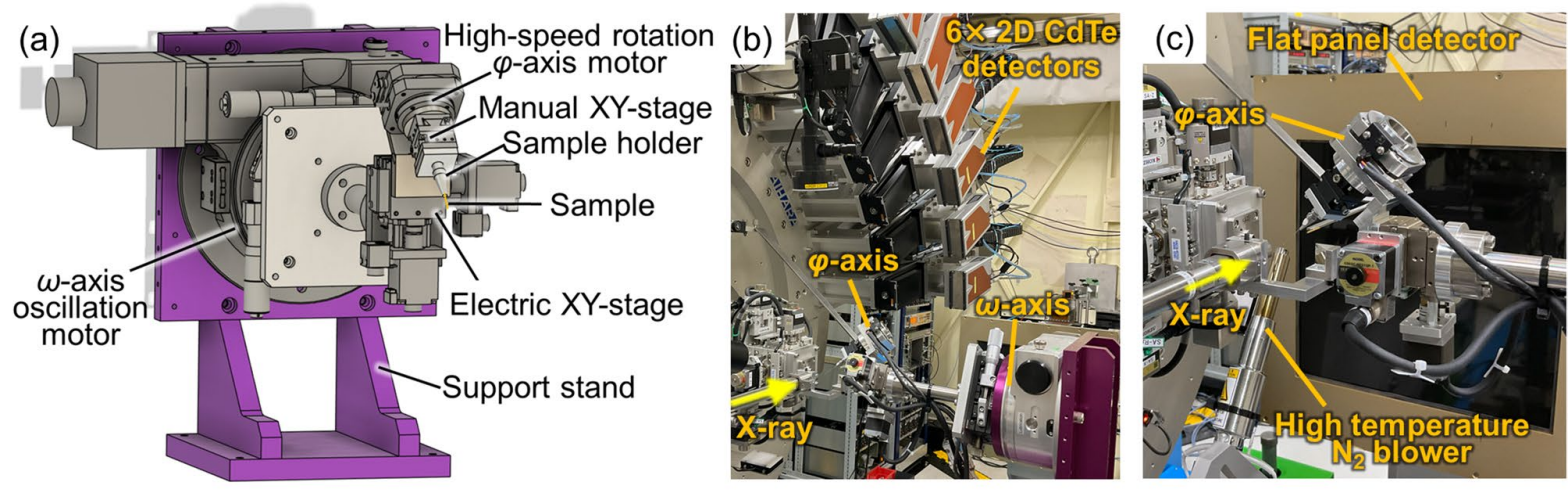
(**a**) Schematic of the two-axis rotation attachment. (**b**,** c**) Photographic images of the measurement layout using the two-axis rotation attachment with (**b**) six sets of 2D detectors and (**c**) a flat panel detector.

The two-axis rotation attachment employed herein is shown schematically in Fig. 1a. This attachment consists of two rotational axes, namely the *ω*-axis, which is oriented perpendicular to the X-ray beam, and the *φ*-axis, which is tilted by 45° relative to the *ω*-axis. The *φ*-axis is driven by a high-speed stepping motor capable of continuous rotation at speeds of ~1200 rpm. The *ω*-axis stage is capable of operating over ±180°. It also operates in continuous motion and is configured to scan over an angular range slightly exceeding 180°, with PXRD data acquired only within the 180° region where the angular velocity remains constant. The combination of continuous *ω*-axis oscillation and *φ*-axis rotation leads to more uniform sampling of the reciprocal space. A manual XY positioning stage is mounted on the *φ*-axis, while an electric XY stage is mounted on the *ω*-axis. These stages facilitate the alignment of the sample position at the intersection between the two axes. The attachment is set on a support stand to easily align with the incident X-ray positions of the powder diffractometers. The support stand equipped with the *ω*-oscillation motor is designed to easily accommodate a wide variety of measurements, such as high-pressure experiments^15^. The support stand can be set on an electric XYZ stage, which allows precise adjustment of the sample position at the X-ray beam position.

Photographic images of the measurement setup and detector arrangement are shown in Fig. 1b and c, respectively. Synchrotron PXRD experiments were primarily conducted using the powder diffraction measurement system installed in the third experimental hutch at BL13XU of SPring-8^14^. PXRD patterns were acquired using either six sets of hybrid photon-counting 2D CdTe detectors (LAMBDA 750k, X-Spectrum GmbH) or a large-area FPD (XRD1611, Varex Imaging). These fast-readout detector systems enabled rapid data acquisition compared with conventional imaging plate systems. Specifically, multiple photon-counting detectors with a sample-to-detector distance of 630.24 mm allowed the acquisition of high-angular-resolution, low-background PXRD data with access to the high-*Q* region. In this study, the PXRD patterns were acquired at four positions to bridge the 2*θ* gaps between the detectors and fill in the 2*θ* gaps within each detector^14^. In contrast, the large-area FPD provided superior particle statistics compared with the 2D CdTe detectors because of its broader integration range. The obtained 2D PXRD images were integrated into one-dimensional (1D) patterns using an in-house Python script based on the pyFAI library^16^. A two-axis rotation attachment is also available at BL02B2 of SPring-8^3^, which is equipped with 1D detectors (MYTHEN, Dectris Ltd.) and an FPD (XRD3025, Varex Imaging).

To enable in situ high-temperature PXRD during melting or crystal growth in flux, capillary samples were mounted on a tilted *φ*-axis spinner. This setup is advantageous because it can stabilize the molten phase position in the capillary under the influence of gravity. During exposure, the *ω*-axis was kept stationary, while the *φ*-axis was rotated at ~ 30 rpm. For bulk or single-crystalline samples, the measurement sample was rotated around the *φ*-axis at ~ 1200 rpm and oscillated around the *ω*-axis over a range of 180° within 30 s. Consequently, the *φ*-axis rotation was significantly faster than the *ω*-axis oscillation, thereby enabling two-axis rotation to achieve sufficient particle statistics. The PXRD data were collected using 2D CdTe detectors by continuously recording 60 frames with an exposure time of 0.5 s per frame, corresponding to 3° rotation around the *ω*-axis per frame. This frame-by-frame acquisition approach is advantageous for indexing overlapping Bragg peaks. Thus, combining a two-axis rotation attachment with fast area detectors facilitates the construction of synchrotron PXRD layouts tailored to specific applications.

### Powder diffraction patterns of liquid and solidified Zn

**Fig. 2:**
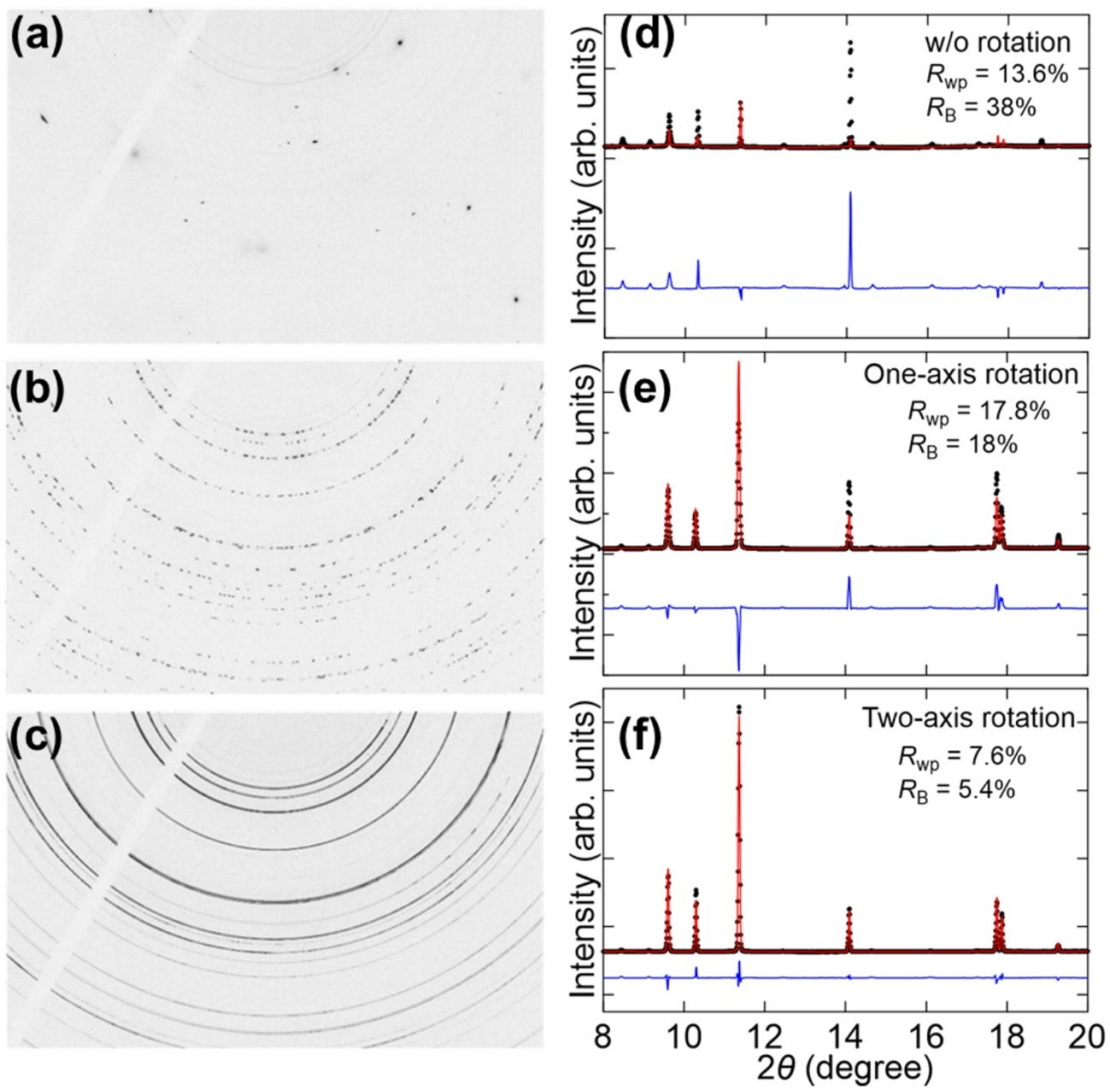
(**a–c**) Two-dimensional XRD patterns recorded for Zn at RT after solidification, collected using the FPD at BL02B2: (**a**) Without sample rotation, (**b**) with one-axis rotation, and (**c**) with two-axis rotation. (**d–f**) Corresponding 1D PXRD patterns derived from the patterns shown in panels (**a**–**c**), together with their Rietveld refinement results: (**d**) Without sample rotation, (**e**) with one-axis rotation, and (**f**) with two-axis rotation. Black dots, red solid lines, and blue solid lines represent the observed PXRD data, calculated patterns, and differences between them, respectively. Measurements were performed using 30 keV X-rays.

To evaluate the performance of a Gandolfi-type two-axis rotation attachment, PXRD patterns of solidified Zn samples were collected. Figure 2a–c present the 2D XRD patterns of Zn recorded at room temperature (RT) using the FPD detector, following melting and subsequent solidification. As shown in Fig. 2a, the 2D XRD pattern collected at RT without capillary rotation consists of single-crystal spots, along with minor contributions from oxidized Zn. This indicates that, after solidification, the sample grew into single-crystalline grains; thus, it was difficult to obtain PXRD patterns with sufficient particle statistics. When the capillary sample was rotated using a one-axis spinner, the number of observable spots significantly increased, yielding discrete Debye rings. Further measurements using a two-axis rotation attachment improved the uniformity of the Debye rings, producing nearly continuous rings, although some inhomogeneities remained. Figure 2d–f show the 1D PXRD patterns obtained from the 2D XRD data, together with the Rietveld analysis results. Employing two-axis rotation significantly improved both *R*_wp_ and *R*_B_ values. Specifically, *R*_wp_ and *R*_B_ values of 13.6% and 38%, respectively, were obtained without rotation. With one-axis rotation, the corresponding values were 17.8% and 18%. In contrast, two-axis rotation reduced these values to 7.6% and 5.4%, respectively. These results confirm that PXRD data with sufficient particle statistics can be obtained using the two-axis rotation mechanism, even for solidified samples.

**Fig. 3:**
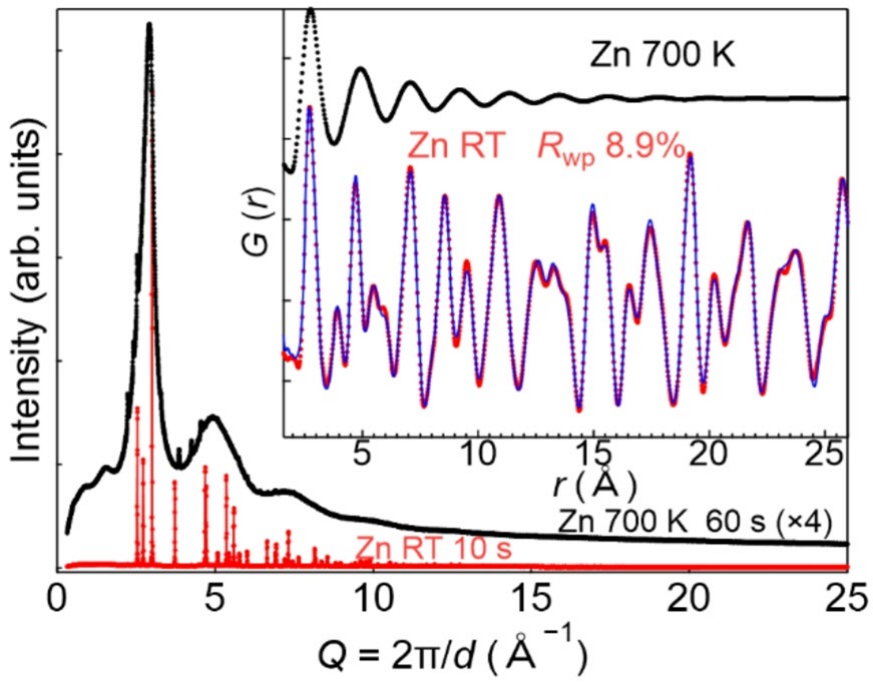
PXRD patterns collected for Zn at RT (red) and 700 K (black). For clarity, the intensity of the PXRD pattern at 700 K is plotted with a scaling factor of four, and the exposure time is longer than that for the RT measurements. The inset shows the reduced *G*(*r*) of Zn at RT and 700 K. For the RT data, the calculated PDF fitting results are shown in blue. Measurements were performed using 60 keV X-rays at BL13XU.

The capability of the tilted *φ*-axis spinner configuration was demonstrated by PXRD measurement of Zn at RT and 700 K, which is above the melting point of Zn. Figure 3 shows PXRD patterns collected for Zn at RT and 700 K. The PXRD pattern recorded at RT was predominantly indexed to a hexagonal Zn structure (*P*6_3_/*mmc*)^17^, with refined lattice constants of *a* = 2.66460(1) Å and *c* = 4.94718(4) Å. A minor contribution from the ZnO phase was also observed, which was estimated to be ~ 1 wt% based on Rietveld analysis^18,19^. The reduced *G*(*r*) of Zn at RT, together with the fitting results are shown in the inset of Fig. 3. The observed *G*(*r*) was well reproduced by the calculated PDF patterns considering the hexagonal Zn structure, yielding a reliable weighted profile *R*-factor (*R*_wp_) of 8.9%. Notably, the features of *G*(*r*) were similar to those of previous synchrotron PXRD results^20^, indicating that reliable PDF analysis can be achieved even when using a tilted *φ*-axis spinner configuration.

As shown in Fig. 3, the PXRD pattern recorded at 700 K exhibited smooth and broad features, indicating that Zn entered the liquid phase. Minor diffraction peaks from ZnO were also present, similar to those observed in the RT data. The obtained 1D PXRD data were smooth and showed no discontinuities, indicating that variations in the liquid sample position were negligibly small even during multi-position PXRD acquisition and subsequent gap interpolation. The reduced *G*(*r*) values are presented in the inset of Fig. 3. The strongest *G*(*r*) peak appears at ~ 2.76 Å, corresponding to nearly twice the atomic radius of Zn, while being slightly larger than the corresponding peak obtained for solid Zn at RT (2.70 Å)^21^. The second and third strongest peaks can be observed at ~ 4.92 and ~ 7.05 Å, respectively. These results are consistent with those of previous studies^22^, demonstrating that the tilted configuration effectively stabilizes the position of the liquid phase within the capillary during measurements.

### In situ observation of the synthesis of LiCoO_**2**_ in a molten salt

To obtain high-performance LiCoO_2_ electrode materials, various synthetic methods have been developed, including solid-state reactions, sol–gel approach, hydrothermal synthesis, and flux methods^23–26^. For example, flux synthesis enables the formation of LiCoO_2_ at relatively low temperatures and yields materials with high specific capacity^23,24,27^. To demonstrate the in situ observation of crystal growth from a liquid phase using the tilted spinner system, the synthesis of LiCoO_2_ in a LiCl–LiNO_3_ molten flux was investigated. One key difficulty associated with the in situ observation of this process lies in the movement of both the sample and flux within the capillary once the LiCl–LiNO_3_ mixture becomes molten, which can impede the detection of intrinsic phase changes. This issue was addressed by employing a tilted spinner configuration, which stabilized the sample position during heating and enabled a more reliable in situ observation of the crystallization behavior.

**Fig. 4:**
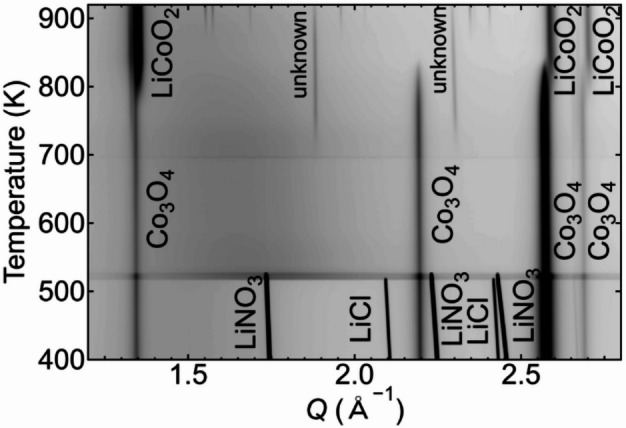
PXRD intensity maps for the synthesis of LiCoO_2_. The PXRD data were measured with the FPD using 25 keV X-rays during heating at BL13XU.

Figure 4 shows the temperature dependence of the PXRD patterns recorded for the Co_3_O_4_ and LiCl–LiNO_3_ mixtures during heating. The PXRD patterns were subsequently transformed into a 2D PXRD intensity map, correlating *Q* with the temperature. At 400 K, the observed diffraction peaks were indexed to the LiNO_3_, LiCl, and Co_3_O_4_ phases. The diffraction peaks of LiCl disappeared at temperatures > 517 K, consistent with the melting point of the eutectic mixture of LiNO_3_ and LiCl^24,28^. Additionally, the diffraction peaks of LiNO_3_ disappeared above 528 K, which is slightly below the melting point of LiNO_3_. Beyond this temperature, the background level increased owing to the generation of the liquid phase, whereas the Co_3_O_4_ phase remained clearly observable. These results indicate that both the crystalline Co_3_O_4_ and liquid phases can be stably observed, even in the presence of the melt, although slight fluctuations in the PXRD intensity were observed just above the melting point, possibly owing to the density changes associated with melting.

After flux melting, distinct changes were observed in the Co-based crystalline phases. Above 710 K, weak unidentified diffraction peaks appeared, whereas the intensity of the peaks indexed to the LiCoO_2_ phase increased at temperatures > 790 K. Correspondingly, the intensity of the diffraction peaks corresponding to the Co_3_O_4_ phase gradually decreased. Above 835 K, the diffraction peaks attributed to Co_3_O_4_ disappeared. Accordingly, the peaks associated with LiCoO₂ became dominant, although slight splitting of a diffraction peak attributable to the LiCoO_2_ phase was observed at ~ 1.3 Å^−1^, suggesting the presence of multiple LiCoO_2_-related phase. The in situ PXRD measurements confirmed that LiCoO_2_ was the predominant crystalline phase. These results confirm that the tilted spinner configuration developed herein enables the reproducible in situ observation of crystalline phase evolution within the molten flux.

## High-resolution PXRD data collection for the crystalline samples

Since orientation averaging via two-axis rotation enables PXRD data collection from limited or non-powdered specimens, Gandolfi cameras are often useful for obtaining PXRD patterns of extraterrestrial samples and terrestrial minerals^7,11,12^. This study aimed to acquire 1D PXRD data with short exposure times, high angular resolution, and minimal peak overlap using a longer sample-to-detector distance than conventional Gandolfi cameras, while also leveraging the benefits of fast area detectors. San Carlos olivine, a material that is commonly used in experimental petrological studies, was employed to facilitate these measurements. The chemical composition of the olivine crystals from this locality has been previously reported as (Mg_0.9_Fe_0.1_)_2_SiO_4_^29^.

**Fig. 5:**
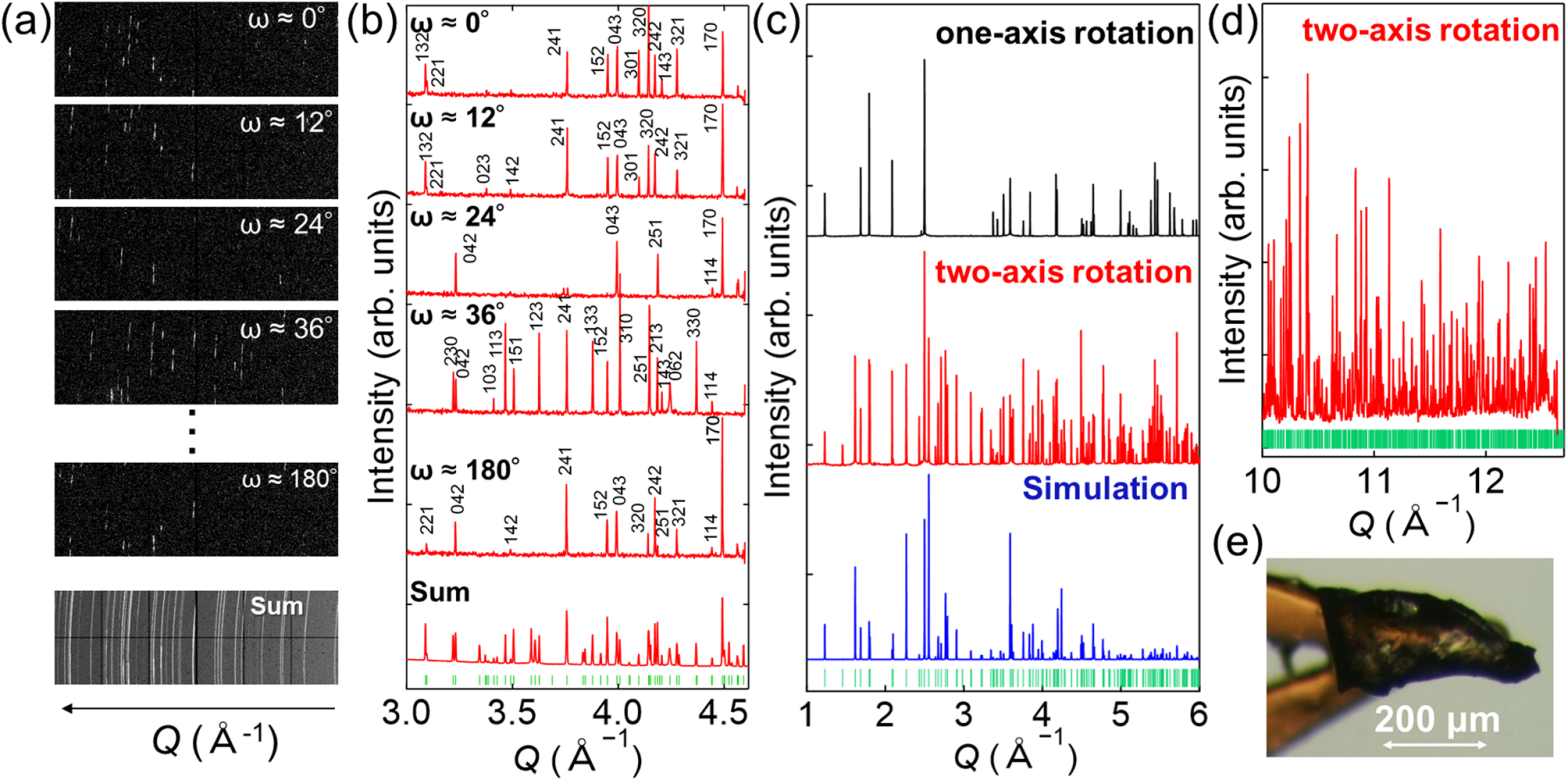
(**a**) 2D XRD frames collected at a fixed 2*θ* angle using 2D CdTe detectors and (**b**) the corresponding 1D PXRD patterns obtained using the two-axis rotation system. Each dataset covers a total Δ*ω* of ~3°, with representative frames being recorded at different *ω* angles. A summed image of all frames and the resulting 1D PXRD pattern are also presented. Miller indices of the observed diffraction peaks are labeled on the 1D data, and the green ticks mark the reflection positions of olivine. (**c**) PXRD patterns measured with one-axis (black) and two-axis (red) rotation, together with the simulated profiles (blue) and (**d**) magnified image of the high-angle region collected using the two-axis rotation attachment. The background signal was subtracted from the observed PXRD patterns to facilitate clearer interpretation of the diffraction peaks. Measurements were performed using 25 keV X-rays at BL13XU. (**e**) Photographic image of the olivine crystal used for measurement.

Figure 5a shows representative sections of the 2D XRD image recorded for olivine. Each frame corresponds to data collected during a full 360° rotation of the *φ*-axis and an ~ 3° rotation of the *ω*-axis, with the *ω*-angle being incrementally shifted between frames. Single-crystalline diffraction spots were observed, and their positions varied between frames. Accordingly, as shown in Fig. 5b, converting each 2D pattern into a 1D profile yielded XRD peaks that varied from frame to frame. This frame-by-frame 1D data allowed indexing with reduced peak overlap compared with that observed for the summed data. Furthermore, the high angular resolution of the XRD data facilitated the indexing of complex crystal structures. As shown in the lower images of Fig. 5b, the majority of the expected diffraction peaks for the olivine *Pnma* structure are clearly observed in the summed data, indicating the feasibility of structural determination.

Figure 5c shows the PXRD pattern and simulated profile. The broad background signal, originating primarily from Kapton and air, was subtracted from the observed spectrum. As shown in Fig. 5d, clear XRD peaks were observed even in the *Q* ≈ 12 Å^−1^ region, which cannot be accessed with conventional Cu *Kα* radiation, highlighting the advantage of high-energy synchrotron X-rays. Additionally, the use of high-angle PXRD data is advantageous for determining accurate lattice constants. Le Bail analysis^30,31^ yielded lattice parameters of *a* = 10.22168(3) Å, *b* = 5.99088(2) Å, and *c* = 4.76040(1) Å for olivine.

Figure 5c compares the PXRD data obtained using the two-axis rotation system with those obtained using a conventional one-axis rotation system. Focusing on the region of *Q* < 3 Å^−1^, seven peaks were observed with the one-axis rotation system, corresponding to approximately one-third of the 22 peaks predicted in the simulated pattern. In contrast, 21 diffraction peaks were clearly detected using the two-axis rotation system, in close agreement with the simulation results. Notably, the 411 reflection at *Q* ≈ 2.98 Å^−1^ was not clearly observed with the two-axis rotation system, possibly owing to its low intensity and/or crystal orientation, whereas it was weakly detected in the one-axis rotation data. Although certain reflections could be observed only in the one-axis rotation data owing to differences in crystal orientation during measurement, the two-axis rotation system yields a substantially larger number of observable reflections overall, indicating improved sampling of reciprocal space and more frequent fulfillment of diffraction conditions. Notably, the entire measurement performed using the two-axis rotation system was completed by acquiring 30-s exposures at only four different 2*θ* positions, enabling high-precision data collection within a short timeframe.

Employing a fast-readout 2D detector with a long sample-to-detector distance enables efficient high-resolution XRD data collection within a short measurement period. By exploiting a short measurement time, high-throughput PXRD pattern acquisition becomes feasible even for minute crystals that are difficult to pulverize. This approach enables the straightforward characterization of systems in which oxidation occurring during pulverization or mechanical processing alters the intrinsic structural state, including sintered permanent magnet materials containing metallic secondary phases susceptible to oxidation and alloy materials whose structure and crystallinity can be readily modified by grinding^32–35^. In addition, a two-axis rotation system is expected to facilitate high particle-statistics PXRD data acquisition during the growth of semiconductor materials as well as during transformation processes, such as martensitic transformations involving minor crystalline phases in alloy materials. Consequently, the system developed in this study is applicable not only to materials that are obtainable only as microcrystals, such as minerals, but also to practical materials that require structural characterization without pulverization, including metallic materials and commercially fabricated textured bulk samples.

## Conclusion

A high-performance synchrotron PXRD technique was developed using a two-axis rotation attachment to achieve improved particle statistics within a short timeframe. By implementing a high-speed *φ*-axis spinner and *ω*-axis rotation, the system enabled efficient averaging over crystal orientations, facilitating reliable and reproducible data acquisition, even from limited or coarse-grained samples. Specifically, integrating the two-axis rotation system with a high-speed spinner and fast area detectors enabled sub-second continuous imaging and frame-by-frame peak separation, thereby facilitating efficient indexing of complex PXRD data. In addition, the use of a 45°-tilted spinner effectively stabilized the sample position of the melting phases in the capillary, enabling reproducible in situ observations of the crystallization processes and analysis of the PDF. These features demonstrate the versatility of the developed system and its potential applicability to a wide range of samples. Finally, by exploiting the ability to obtain PXRD patterns from unpulverized samples within a short measurement time, we are currently investigating crystal structure analyses of oriented materials such as steels and magnets. The results of these studies will be presented in due course.

## Methods

For in situ heating experiments designed to observe the melting processes, an incident X-ray beam with a size of 0.2 mm (vertical) × 0.5 mm (horizontal) was used at the X-ray diffraction and scattering I beamline (BL13XU) of the SPring-8 facility. During data acquisition, the *ω*-axis was kept stationary, while the *φ*-axis was rotated at ~ 30 rpm. The temperature was controlled using hot N_2_ gas blowers that were permanently installed on the beamline^3,14^. A PDF analysis was performed on molten Zn (5 N, Nilaco), which was sealed in a 0.2-mm borosilicate glass capillary under an Ar atmosphere. The measurements were conducted at RT and 700 K. PXRD patterns were recorded using multiple 2D CdTe detectors with exposure times of 60 s at 700 K and 10 s at RT. The incident X-ray energy was set to 60 keV (0.2069 Å), and the maximum value of *Q* (*Q*_max_) was ~ 35 Å^−1^. The reduced PDFs (*G*(*r*)) were calculated using Gudrun software^36^, and the PDF fits were obtained using PDFgui software^37^. The 2D XRD patterns of the solidified Zn were subsequently collected using the FPD at RT with 30 keV X-rays (0.4133 Å) at BL02B2. To demonstrate the usefulness of the two-axis rotation attachment, reference data were collected under non-rotating conditions and also in the single-axis rotation mode on a spinner stage permanently installed at BL02B2, with the rotation axis oriented perpendicular to the X-ray beam. Rietveld analyses were performed using TOPAS software^31^.

As a demonstration of crystal growth observation from the liquid phase, the synthesis of the LiCoO_2_ phase was investigated, with starting materials selected based on previous reports^23,27^. A flux of pre-dried LiNO_3_ (2 N up, Kojundo Chemical Laboratory) and LiCl (3 N, FUJIFILM Wako Pure Chemical) was employed in a molar ratio of 0.88:0.12. Co_3_O_4_, which was prepared via thermal decomposition of Co(NO_3_)_2_ 6H_2_O (3 N up, Kojundo Chemical Laboratory), served as the cobalt source. The molar ratio of the mixed salts to Co was 4:1, and mixing was performed in an Ar-filled glove box. The mixture was placed in a quartz capillary with a diameter of 0.7 mm, and heating was conducted from RT to 920 K at a heating rate of 10 K/min. PXRD patterns were recorded using the FPD with an exposure time of 2 s. The incident X-ray energy with a size of 0.2 mm (vertical) × 0.5 mm (horizontal) was set to 25 keV (0.4960 Å).

For bulk samples with coarse crystal grains, a larger beam size of 0.5 mm × 0.5 mm was employed, and the incident X-ray energy was set to 25 keV (0.4960 Å). Each sample was mounted on a Kapton film. To demonstrate the effectiveness of the two-axis rotation system, reference data were collected in the single-axis rotation mode on a six-axis spinner stage that was permanently installed on a high-resolution powder diffractometer at BL13XU, with the rotation axis positioned perpendicular to the X-ray beam.

## Data Availability

Data supporting the findings of this study are available from the corresponding author upon request.
